# Enhancing Apple Cultivar Classification Using Multiview Images

**DOI:** 10.3390/jimaging10040094

**Published:** 2024-04-17

**Authors:** Silvia Krug, Tino Hutschenreuther

**Affiliations:** 1 Department of Computer and Electrical Engineering, Mid Sweden University, Holmgatan 10, 851 70 Sundsvall, Sweden; 2 System Design Department, IMMS Institut für Mikroelektronik- und Mechatronik-Systeme Gemeinnützige GmbH (IMMS GmbH), Ehrenbergstraße 27, 98693 Ilmenau, Germany; tino.hutschenreuther@imms.de

**Keywords:** apple cultivar recognition, deep learning, multiview classification

## Abstract

Apple cultivar classification is challenging due to the inter-class similarity and high intra-class variations. Human experts do not rely on single-view features but rather study each viewpoint of the apple to identify a cultivar, paying close attention to various details. Following our previous work, we try to establish a similar multiview approach for machine-learning (ML)-based apple classification in this paper. In our previous work, we studied apple classification using one single view. While these results were promising, it also became clear that one view alone might not contain enough information in the case of many classes or cultivars. Therefore, exploring multiview classification for this task is the next logical step. Multiview classification is nothing new, and we use state-of-the-art approaches as a base. Our goal is to find the best approach for the specific apple classification task and study what is achievable with the given methods towards our future goal of applying this on a mobile device without the need for internet connectivity. In this study, we compare an ensemble model with two cases where we use single networks: one without view specialization trained on all available images without view assignment and one where we combine the separate views into a single image of one specific instance. The two latter options reflect dataset organization and preprocessing to allow the use of smaller models in terms of stored weights and number of operations than an ensemble model. We compare the different approaches based on our custom apple cultivar dataset. The results show that the state-of-the-art ensemble provides the best result. However, using images with combined views shows a decrease in accuracy by 3% while requiring only 60% of the memory for weights. Thus, simpler approaches with enhanced preprocessing can open a trade-off for classification tasks on mobile devices.

## 1. Introduction

In Germany alone, over 1500 apple cultivars are known, and the number is much higher worldwide. Most of these cultivars have been grown commercially in the past but have been superseded in productive orchards by 10–25 common cultivars that are grown worldwide. The more traditional cultivars remain as part of historic orchards or forgotten woody meadows. However, their value is still high since the traditional cultivars provide genetic variability and are typically well adapted to local climate and other environmental conditions [1,2,3]. In addition, they are robust against several diseases. It is, therefore, desirable to collect and secure the existing cultivars.

Mapping and collecting of cultivars is often done in old orchards where experts try to identify possibly rare cultivars and then secure them. These are then often added to collections if correctly identified. This requires in-field classification, ideally directly at the tree in question, to avoid confusion of probes. Securing cultivars is carried out as part of different genetic resource collections (e.g., [3,4]). In the case of apples, this means several orchards with living trees, as growing trees from seeds would result in new cultivars. The correct cultivar classification within the collections is also important and requires constant updating whenever trees must be replaced. The reason for this is potential errors when collecting and handling the material during the required crafting and replanting [5].

In both cases, the review of cultivars in a collection and the in-field mapping, the classification is carried out by human experts based on fruit. Experts use several fruits at an optimal ripeness state to classify them mainly based on experience and so-called descriptors for phenotypic cultivar characteristics [6]. They request multiple fruits to initially identify whether the fruit in question shows typical characteristics. Afterward, the most typical fruit is assessed in detail from the outside and inside using a longitudinal cut through the fruit. This essentially represents a multiview approach.

To minimize the risk of errors during probe collection, a mobile tool to support field workers with the classification would be ideal. It should work without the requirement to upload images directly to a cloud service for classification. Our goal is to build such a tool in the future. To achieve this, it is important not only to classify the cultivar correctly but also to do it in a resource-efficient way. The main challenge, however, is to achieve good classification results in the first place. Since different apple cultivars are similar while the variability of the apples from one cultivar is high, few features define whether it is one or the other cultivar. In our previous work [7], we showed that it is promising to use machine-learning (ML) and deep neural networks (DNN) for this task. That work focused on a single view, using only images from the longitudinal cut. Our results showed that even a single view can be used to distinguish cultivars. However, with an increasing number of cultivars, we expect the accuracy of that approach to be reduced since further features visible from the outside of an apple might become more important, according to the experts.

Various works exist that show how to handle such multiview problems for various other applications (e.g., in [8,9]). It is state of the art to employ ensemble methods where networks are trained per viewpoint, and the information becomes combined or fused at some point. Recent studies focus on how and where to fuse the information best [8,9]. However, training sub-models per viewpoint results in higher memory requirements for the overall model, which might hinder its deployment on mobile devices in the field. Cultivar classification is starting to attract more attention for various crops (e.g., apple, hazelnuts [10,11], tomato [12], cherry [13]). These mostly rely on single-view models to classify rather well separable cultivars with only a few classes. This is problematic since too simple approaches will not generalize well for many cultivars with high inter-class similarity and high intra-class variability, as is the case with approximately 1500 apple cultivars. To allow a model to gather more information about fruit, we decided to study state-of-the-art multiview approaches in this paper to evaluate their benefits for cultivar classification. To the best of our knowledge, this is the first study combining multiview classification with cultivar-level classification. At the same time, we keep in mind the final goal of building a tool suitable for execution on mobile devices. To achieve this, the chosen model should be small in terms of required weights and calculations. While state-of-the-art approaches to shrink good models exist (e.g., using pruning as in [14,15]), the focus of this paper is to find the initial model for the cultivar classification problem. We, therefore, pay attention to the model size but do not apply further optimization techniques to shrink the model. We address this by exploring different options to realize classification based on multiview images. The first option is a state-of-the-art ensemble baseline with one model per view and late fusion before the classifier. The two further options use multiple images but employ a single model only. One learns a class from mixed-view images, and the other uses specially constructed images, where one image contains all available views. Based on the previous results, we build our study around the EfficientNet model family [16].

Our contributions are therefore:

- evaluation of three multiview options for fruit cultivar classification to follow human expert
- presentation of a specific dataset for apple cultivar classification using multiple views per fruit
- dataset preprocessing to utilize multiview information without using a true multiview model architecture to reduce model size without applying shrinking techniques
- explore limitations of the cultivar classification approaches.

The remainder of the paper is organized as follows. In the next section, we present state-of-the-art work on multiview classification from other fields, as well as the current work regarding plant and cultivar classification. Afterward, we present the base model structure and training involved in this study. Section 5 shows how the different models perform and adds a discussion of limitations as well as ideas for further enhancements. In Section 6, we finally draw conclusions and highlight the next steps.

## 2. Related Work

Phenology-based recognition and classification of plant species, organs, or diseases have become popular again with the introduction of computer vision ML methods [17,18]. These are highly interesting despite the availability of genetic analyses for species/cultivar classification because classification based on morphological traits could be executed potentially autonomously in the field.

Regarding plant and animal species classification, there are several apps built around computer vision for plants (e.g., [19,20,21]) or other data such as audio-based bird call identification [22,23]. These show the potential of in-field classification and enable citizen-science studies on, e.g., the distribution of certain species in a wider area. Regarding apple cultivars, this citizen-science feature is interesting, too. Today, expensive mapping projects are performed by experts to assess the spatial distribution of different cultivars in various regions to enable preservation work for rare or regionally important cultivars. These projects typically select only a few representative orchards. Here, a field tool could help to reduce costs and thus cover larger areas.

FloraIncognita [21] is an example of such a field tool and enables robust species classification based on different views of plant organs. The required data set consists of multiple images and viewpoints (e.g., focusing on various plant organs) as well as different environmental conditions such as time of day and season. The app is, in the end, asking the user to take photos in a specific order for the classification, where each image represents a different view of the plant in question. It is thus a multiview approach. The same group studied the impact of different information fusion strategies [9], similarly to the work presented in [24]. However, their tool relies on Internet connectivity to upload the images to a server for classification and is not running locally on the device.

Many applications use multiview classification [25] ranging from 3D-shape recognition [26] to more recent focus on vehicle classification [8] over to various applications in the agricultural domain. There the examples include apple classification [27], fruit ripeness assessment [28,29,30], weed classification [31] and general plant assessment [9]. The work focused on plants does try to classify mainly species, while only in [27], the authors focus on cultivar classification using hyperspectral information from the outside view of an apple.

When using multiview classification, it is important to identify how and where to fuse the different images per view and how to handle missing information from certain viewpoints. The first point has already been addressed multiple times, e.g., in [8,9,24,32]. The second is starting to gain attention in the community, e.g., in [33,34] discussing how to handle missing information, while in [35], the authors address how to handle the imbalanced dataset. These are important aspects, and we add some test cases with missing data to our study to observe their impact.

Besides the work in [27], cultivar classification seems to draw more attention. This also shows the importance of cultivar classification. In addition to apples, studies include other fruit such as grapes [36,37], cherries [13], hazelnuts [10,11], or tomatoes [12]. Many works regarding apple cultivars have emerged over the past few years. Table 1 gives an overview of the different ML tools applied, the used datasets, as well as whether they follow an expert approach and use multiview capabilities. Concerning the used ML approaches, Multi-Layer Perceptrons (MLPs) and other Deep Learning (DL) approaches, such as Convolutional Neural Networks (CNNs), are the most common. In addition, some studies use rather traditional ML approaches such as Support Vector Machines (SVMs) or k-nearest Neighbors (kNNs).

In this paper, we follow the general idea of traditional expert assessments as described in [6] and study corresponding views for classification. This is the first study on multiview classification for apples taking this into account. The dataset used is limited but comparable to other small datasets used in the literature.

Since we target a mobile application running the classification locally on the devices, model performance and resource requirements are important. This trade-off currently gains much intention in industrial applications, e.g., in [53]. The approaches to pruning or quantizing the models are state of the art as, e.g., described in [14,15]. In addition, feature selection mechanisms are discussed [54]. In this paper, we will focus on feature selection by studying different input options for the images and different model structures as a first step. If needed, one can evaluate further measures to reduce the model size later.

## 3. Dataset Collection and Preparation

### 3.1. Image Collection

**Figure 1:**
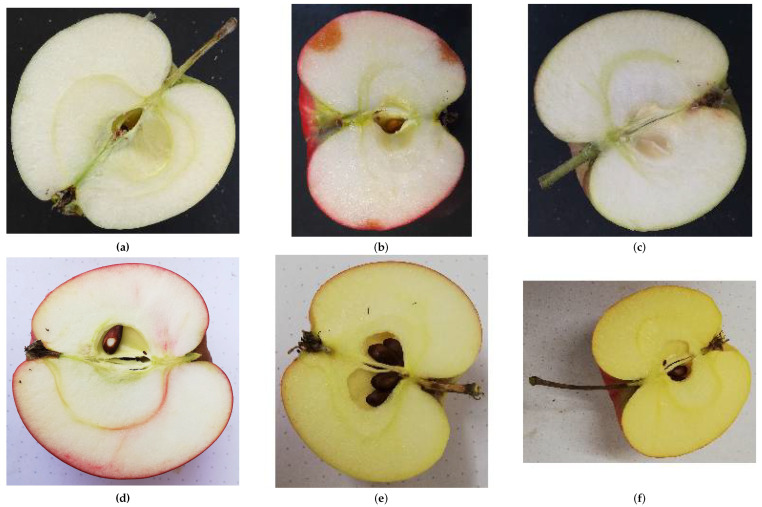
Example images from each class. (**a**) White Transparent. (**b**) Red Astrachan. (**c**) James Grieve. (**d**) Herrenhut. (**e**) Carola. (**f**) Pinova. All images except “Herrenhut” are from [7] provided under CC BY 4.0 license.

For this study, we reuse our custom apple cultivar dataset presented in our previous study [7]. This contains the longitudinal cuts of five apple cultivars as shown in Figure 1. The images except for “Herrenhut” are taken from [7] and are available under the CC BY 4.0 license. Experts classified the apples used as a base for this dataset, and we collected the apples from traditional orchards to ensure realistic images. The apples were collected from 2020 to 2022 and photographed in different light, ripeness states, and with different backgrounds to account for variation. For this study we extended the dataset by one additional class “Herrenhut”.

**Figure 2:**
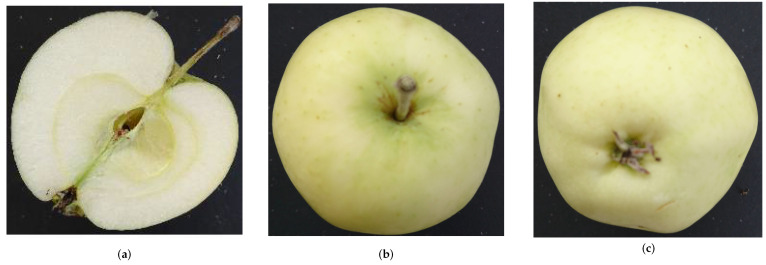
Example images from each view for White Transparent. (**a**) Longitudinal Cut. (**b**) Stem. (**c**) Calyx.

To enable the study of multiview classification, we extend the dataset by adding images from two additional viewpoints per apple. Following the expert approach, we selected the stem and calyx views. For each apple, we thus have up to three images that are named with a unique id for the apple, an identifier for the view v∈1,2,3 as well as a sequential number for each image to allow the identification of a single apple and the corresponding views. Figure 2 shows an example of each view. All images were captured using smartphone cameras.

For some apples, we did not record the images for all views since we originally targeted a model trained on longitudinal cut images only. As a result, we have some apples where one or two views are missing, mostly stem or calyx images. We decided to integrate these apples into our study to assess how the models perform in case of missing information. In total, we collected 2030 images from 789 apples.

### 3.2. Image Preprocessing

Based on the collected images described in the previous section, we then derive three datasets for our study:

1. a dataset where the images from each view are treated as separate channels.
2. a dataset where all images are stored in one folder per class. This mixes all views into one single folder.
3. a dataset using specifically preprocessed images, where the corresponding images of one apple are combined into one image containing all views.

This allows the study of different multiview approaches.

For each individual-view image, we follow the preprocessing approach of our work in [7], where we do not feed the original camera image into the model. We found in that paper that the classification result improves if the shape of the apple does not become skewed by the internal preprocessing of the model that re-scales the image to a square shape. Instead, we manually preprocess the image. This involves cropping the image to a bounding box around the apple and adding buffers to create square images that preserve the original aspect ratio of the apple. The same approach is used for the stem and calyx views in this paper.

**Figure 3:**
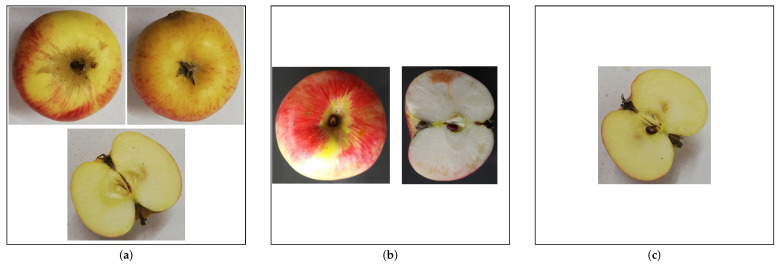
Example images showing the impact of missing data on combined images from class “Carola”. We added a frame around the image to highlight the true extent of each variant. (**a**) Three views. (**b**) Two Views. (**c**) One View.

Similarly, we combine the three views into one bigger square image for dataset three. To do this, we build a script that scans a folder containing all images, finds the views belonging to one apple, and adds them to a new combined image. The individual images are arranged depending on which views are available. Figure 3 shows three resulting example images with only one, two, or all three views available of an apple. We used a frame around each image to highlight the actual dimensions and arrangement of the views.

We are aware that the described preprocessing steps add some overhead to the inference on a mobile device since cropping with aspect ratio is needed in any case. If combined images prove beneficial, these would have to be generated as well. However, we use a sequence of traditional OpenCV functions for this, which seems feasible on mobile devices as well. Once we have a good classification solution, we plan to evaluate if a single shot detector such as Yolo could be used to extract the patches of interest from images and thus serve as a feature extractor for the classifier similar to that described in [37]. That would also allow the identification of whether the model is fed other images than apples and filter these out.

When combining the images, we first use the square crop generated as described in [7], re-scale the cropped image to a certain size (1500 × 1500 pixels), and then combine the resulting images to a new square image with an additional buffer around the individual-view images. We add the intermediate resize step to have about the same information for the number of pixels per view in the combined image. However, if the resulting image is finally resized to the required input size of the model, this results in fewer pixels per view compared to the other dataset variants. To compensate for this potential information loss, we perform tests with different larger image sizes for this variant, as long as the resulting model is smaller than the ensemble model.

## 4. Classification Method

### 4.1. Model Selection

Our previous study [7] showed that the EfficientNet family [16] of CNN models is the best option for this task. EfficientNetB3 provided good classification accuracy while also offering a compact model design, resulting in a small size. In this paper, we reuse this model family to build our target models.

Overall, we build two general variants: default EfficentNet models and an ensemble model built based on EfficientNet. The first variant is a base EfficientNet model with the custom classifier on top. This is the same setup as in our previous work. In addition to the original EfficientNetB3 variant used in [7], we also explore the EfficientNetB4 and EfficientNetB5 variants. These are tuned for larger input image sizes, and our hypothesis is that using larger images can compensate for the loss of detail, resulting in the combination of three images into one for Dataset 3. The model trained on Dataset 3 represents early input fusion, where the actual fusion is carried out as part of the preprocessing. The model trained on mixed images does not contain a specific fusion mechanism. This variant rather must learn the different views independently, putting more emphasis on the actual classification layers that must learn that multiple views belong to the same class.

As the second model architecture, we use EfficentNetB3 as a base to train one model per view and then add a custom classifier, combining the results from the individual models and creating a traditional ensemble approach. This corresponds to a late fusion strategy or score fusion.

For all cases, we follow a traditional transfer learning approach, using the base model with weights from ImageNet [55] and train a custom top classifier on our apple data, including fine-tuning of the top EfficientNet layers after the initial transfer learning of the classifier. Our custom classifier consists of an AveragePooling Layer, a Dense Layer with ReLU Activation with 1024 neurons, a Dropout Layer, and another final Dense Layer with SoftMax activation as classifier. This classifier is used for all models. We build our models using Keras [56] with a TensorFlow backend [57]. Table 2 lists the models used in this study as well as their characteristics, such as model size, input size, and the dataset used for each model. The characteristics were taken from the Keras website https://keras.io/api/applications (accessed on: 26 February 2024) for the non-ensemble models and estimated for the ensemble variant.

### 4.2. Model Training

Since our previous results showed that carefully constructed datasets allow the model to take the variability of the data into account and provide better results, we decided to follow this approach for this paper as well. As a result, we do not perform cross-validation in this case.

Instead, we train each model variant with five different random number seeds, resulting in five independent training runs, and calculate the relevant metrics accordingly. The models are trained using the Adam optimizer.

During training, we use a checkpoint callback to save the weights of the best epoch with respect to the minimum validation loss. The weights of this epoch are then used to evaluate the model on an unseen test data set.

Each model is trained for a maximum of 50 epochs with early stopping. The training was performed on a Lenovo T14s notebook equipped with an NVIDIA T500 GPU. TensorFlow and Keras were using the GPU when training.

Since our datasets are limited with respect to the data amount, we use data augmentation to mitigate this. We apply only those augmentation options from the Keras API that preserve the image aspect ratio. These are rotation, zooming, and shifting.

We split our data into three separate sections: training, validation, and test. The first two are used during training, while the latter is kept out of the training process to allow an assessment of model performance on truly unseen data. We used 1368 images for training, 360 for validation, and 302 for testing. As mentioned before, the dataset contains images with some variation in ripeness, lighting, and image background. To account for these, we carefully build the dataset so that examples of each case are represented in each subset. We use the same split across all datasets and model combinations. In the case of combined images, the numbers are lower as up to three images result in a new one.

## 5. Results and Discussion

**Figure 4:**
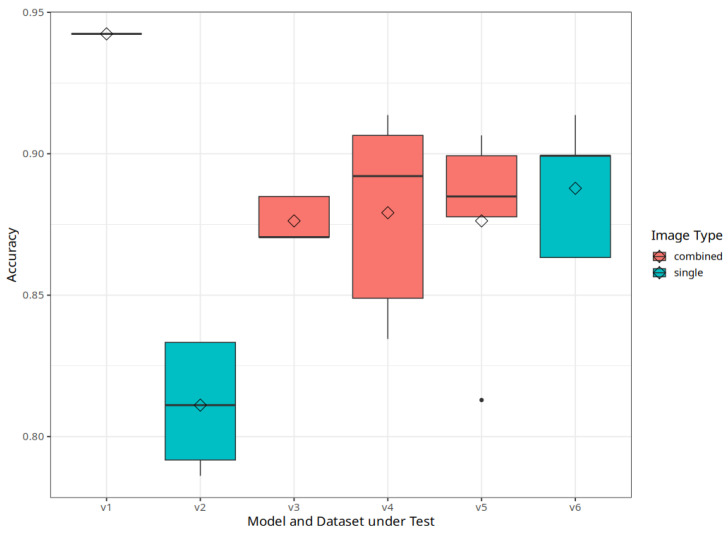
Boxplots of the accuracy score of five independent training runs per model variant on the unseen test data. Model Variants according to Table 3.

Regarding our goal to enable apple cultivar classification, we first analyze the classification performance of the model and dataset combinations under test. Figure 4 and Table 3 show the comparison of the models under test as well as the original EfficientNetB3 model trained in [7].

This shows that the ensemble model can outperform the other variants with the best overall accuracy of 94%. This was expected, as an ensemble model is the state-of-the-art approach for this type of problem. However, the other variants do not perform significantly worse. The only exception is the model trained on Dataset 2, where all images are placed into one folder. This combination shows the worst overall performance. Apparently, the model struggles to learn the correct classes from multiple mixed views, at least with our limited image amount. While this is the simplest approach to handling multiview data, it does not provide good performance on the current dataset.

More interesting is that our previous solution with a model using the one-view dataset shows the same performance as the EfficientNetB3 and EfficientNetB5 variants using dataset 3. Only the EfficientNetB4 Variant shows an improvement over the non-multiview approach.

These results are in line with results from similar apple recognition approaches in the literature, where a classification accuracy of 90% and above is typically achieved (e.g., in [43,52]). However, these results are obtained partially on simpler datasets with fewer cultivars and distinct features to distinguish the classes and are therefore not directly comparable.

**Figure 5:**
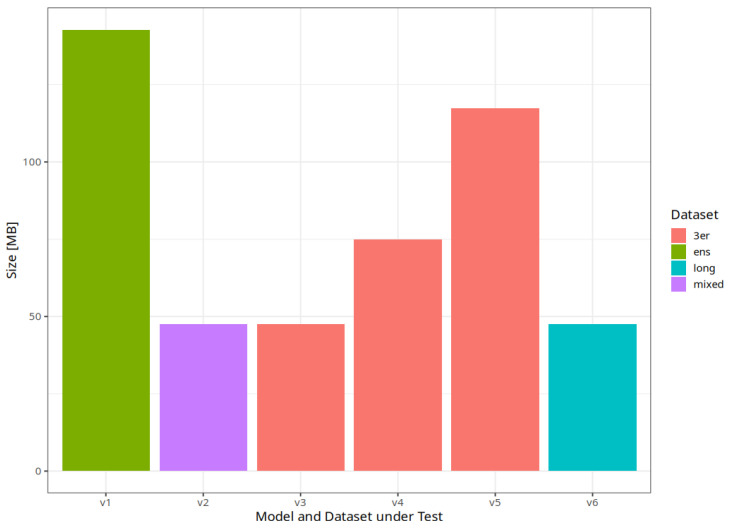
Comparison of model sizes with highlights on the different dataset options.

To be able to run the models on a mobile device, we aim to start with small models before applying shrinking techniques in the future. In our previous work, EfficientNet provided the best results while requiring less memory for model weights [7]. The same base model is used in this work on multiple dataset variants to keep the small model size. In addition, we study three more architectures that, however, result in larger memory requirements in terms of model weights. As a result, we will analyze the trade-off between model performance and size next, and we want to discuss which approach is desirable to further exploit for a mobile classification tool without the need for internet access in the field. Mode size plays a vital role in this. Figure 5 compares the size of the stored model weights graphically and highlights the used dataset in addition. Each EfficientNet model is the same size, irrespective of the dataset used. Therefore, the three base variants are the same here.

We want to achieve the smallest model size possible while achieving good model performance. With respect to these requirements, the ensemble approach is best in terms of accuracy but is also the largest model. Therefore, it may not be the best choice.

Compared to the other options, the EfficientNetB3 variant from our previous work has the best performance of the smaller models. The EfficientNetB4 variant trained on combined images is slightly larger and shows a better average accuracy. With respect to our goal of a small initial model with good classification accuracy, this would suggest going further with the one-view variant, as the performance is comparable. However, we suspect that this does not hold as more cultivars are added, and the additional information from the outer views becomes more important to decide for one class or the other.

When looking at the models using the combined images, the hypothesis that the combination leads to decreased information per view and, thus, a lower accuracy holds, as well as the expectation that an increased input image size can mitigate the accuracy loss. The high variability in the accuracy results suggests that more data and higher quality data during training, especially validation, are needed to allow models to differentiate the classes clearly. This is important since we target an application with many further cultivars, and this gives valuable insights for future data acquisition and preparation.

**Figure 6:**
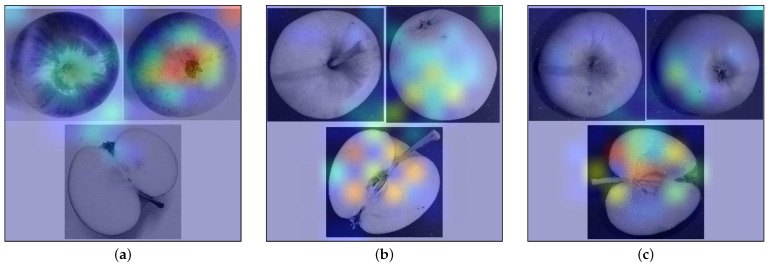
Example images with class-specific attention heatmap overlay for the given input image, highlighting different views used during classification. (**a**) Carola, correctly classified with attention on the outer views. (**b**) Pinova was wrongly classified as White Transparent, with attention paid to calyx and cut. (**c**) Another Pinova, wrongly classified as White Transparent, with attention mainly on the cut view. The original image is shown in grayscale for illustration purposes.

To further study the impact of multiple views and analyze the model errors, we implemented a GradCam visualization to evaluate the model attention of our trained EfficientNetB4 model. Figure 6 shows some selected examples of correctly and incorrectly classified images by model and dataset variant v4. The attention shown represents the class with the highest prediction score.

This shows that the model actually uses information from each view for classification and combines different views as expected. For the first apple, emphasis is placed on both stem and calyx views, while for the other two, it is more on the calyx and longitudinal cut. The first apple is correctly classified, and interestingly, the emphasis shown by the model here corresponds to the areas an expert would focus on when assessing the physical apple. This highlights again the potential of the presented approach for in-field classification.

The wrongly classified images are taken from early fruits that are actually more similar to the White Transparent class than their actual Pinova class. This highlights the importance of good-quality data collection because the variation with different ripeness states caused a misclassification in this case, as the fruits do not show their typical characteristics. This needs further investigation as there are multiple similar effects, e.g., cultivars where only a certain ratio of fruits show a distinct feature, but a lack of that feature may not result in misclassification.

**Figure 7:**
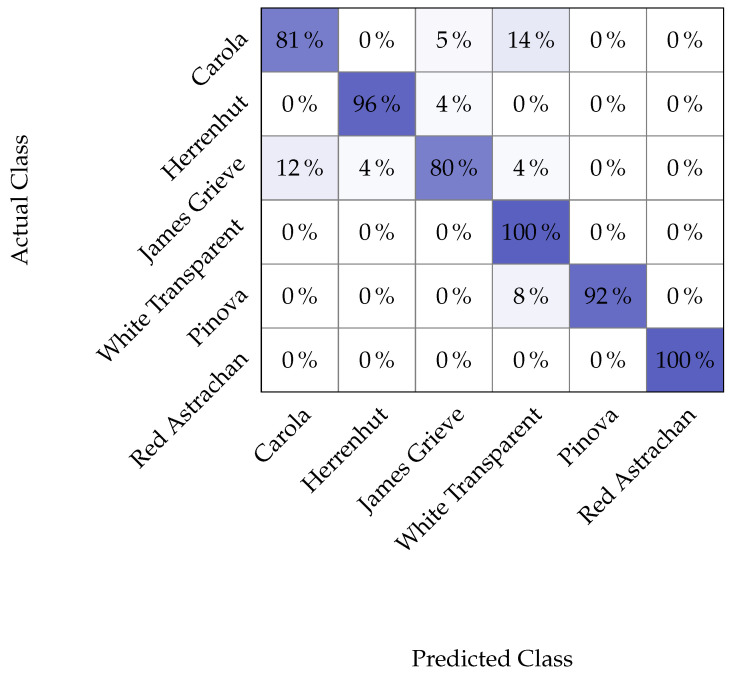
Confusion matrix of the best EfficientNetB4 model under test on Dataset 3.

To analyze the error further, we take a closer look at the confusion matrix of the best EfficientNetB4 model. The confusion matrix is presented in Figure 7.

In total, 12 images were misclassified out of 139 images in the unseen test set. Interestingly, this does not target images with missing views. Therefore, we can assume that the model is able to handle missing data to some extent. Instead, the image quality seems to be more important. Similar to our previous result in [7], the most difficult classes are Carola and James Grieve, which are apparently hard to differentiate based on the available input images. This was already present in our original study using the cut images, which confirms the previous finding. It was, however, surprising that the addition of further views did not allow for a better separation between the two classes. We, therefore, took a closer look at the collected images and found that many are not typical for the given class or show other problems, such as a blur due to the focus being off. In addition to this, we observe the described confusion around White Transparent. This class is in itself classified correctly, but several additional apples from other cultivars are wrongly classified as White Transparent despite belonging to another class. As the grad cam shown in Figure 6 suggests, these are unripe apples that show familiarity with this White Transparent since the cultivar-specific characteristics are not yet properly developed.

We assume that these challenges are not model-related but rather are limitations of the current dataset. These results indicate that we must revisit our dataset and check for typical characteristic images for further analyses to allow the model to properly pick up the differences. In this case, we might have to limit the dataset to typical apples only, and we must ensure that images from early ripeness stages and potentially late storage stages are properly represented in the training and validation data to allow the models to pick up important details on how an apple looks at different times of the year. This is probably true for each important feature, as well as variations in settings such as the light and background. Distributing images with the latter aspects seems to be a good solution since these did not show any obvious problems when analyzing the wrongly classified images.

The trade-off between model size and performance cannot be answered clearly by this study. Some errors are most likely related to data quality, and with six classes, the dataset remains small. As a result, it is not clear whether a single view could be sufficient or not. However, the grad cam result showed that the models use additional information from the outside, and thus, going forward with the multiview approach seems more promising in the long term for a larger number of supported classes. The idea of combining the input images into one single image as a form of input fusion also seems promising if one can increase the image size to mitigate a possible information loss due to the combination.

Alternatively, one could try to use the ensemble model and apply common data reduction techniques such as pruning or quantization to reduce the model size as shown in [14,15]. However, these approaches will again result in an information loss that might be costly for cases with many different classes with high similarity. Here, the loss in information results from either quantization, where fewer bits are available per channel, or pruning, which removes certain weights from the network. We, therefore, want to focus first on developing a larger dataset with more cultivars and then find an appropriate model before starting the tuning for size.

Since we target cultivar classification, the methods should also be evaluated using further datasets. We believe that our approach is suitable for similar problems as long as an expert would use multiple views to assign a certain class. Depending on the problem or fruit at hand, the relevant views might be different and would require specific images per view, as, e.g., the authors in [12] use images of cherry pits. We plan to extend this study to similar problems and their specific data in the future.

## 6. Conclusions and Future Work

In this paper, we extended our previous work on apple cultivar classification by adding multiview capabilities. The goal is to better mimic the approach of human experts and exploit more phenological features of the apple to be able to handle many similar classifiers. Therefore, we compared three options to introduce multiview data with different data preprocessing model structures and discussed the trade-offs. Our results show that the ensemble models perform best but are large, and a dataset with mixed views performs worst. The intermediate approach to combine the inputs and use a single model with increased input image size shows a good compromise in terms of accuracy and size. However, the results for the candidate did not show a clear benefit over the original single-view model. We suspect that this will show with an increased number of cultivars in the dataset where the additional features become more relevant for the differentiation of individual classes.

We, therefore, plan to extend our work on combined images and single-view models using the longitudinal cut view in two ways as the next step. The first step is to revise the dataset, make sure typical apples are part of the validation, and ensure that all the potential intra-class variance is well represented in the split. In parallel to this effort, we will extend our dataset with more cultivars, each featuring a similar number of images per class, and reevaluate the findings of this paper. The goal here is to identify at which point the multiview approach outperforms the single-view model. We plan to test this in two ways: one is an increase in cultivar number, and the other one goes along with this but involves cultivars that are very similar to each other and are problematic for human experts, too. Such a stress test could reveal the true potential and limitations of each approach. Afterward, we plan to extend this work towards building the mobile field classification tool and apply sophisticated model size reduction techniques.

## Figures and Tables

**Table 1:** Literature Comparison.

Paper	Year	ML Tool	Capture	Images	Cultivars	Plant Organ	Fruit Views	Expert Appr.	Multiview
[38]	2012	SVM	Web	90	2	Fruit	Outside	no	no
[39]	2016	Naive Bayes	Phone	150	3	Fruit	Outside	no	no
[40]	2016	MLP/kNN		90	3	Fruit	Outside	no	no
[41]	2019	LDA	Scanner		25	Seeds		no	no
[27]	2020	Naive Bayes	Spectral	180	3	Fruit	Outside	no	no
[42]	2020	CNN	Camera	12,400	14	Leaf		no	no
[43]	2021	CNN	Scanner		3	Fruit	Outside, Cut	no	no
[44]	2021	SVM	Public data	13,000	6	Fruit	Outside	no	no
[45]	2022	CCN	Public data	7159	14	Fruit	Outside	no	no
[46]	2022	Custom DL	Camera	14,400	30	Leaf		no	no
[47]	2022	CNN	Camera		9	Fruit	Outside	no	no
[48]	2023	kNN, SVM		60	2	Fruit	Outside	no	no
[49]	2023	kNN, SVM, MLP	Camera	5830	10	Fruit		no	no
[50]	2023	CNN		120	6	Fruit		no	no
[51]	2023	CNN		8538	13	Fruit	Outside	no	no
[52]	2024	CNN	Camera	5808	10	Fruit	Outside	no	no
[7]	2023	CNN	Phone	600	5	Fruit	Cut	yes	no
ours	2024	CNN	**Phone**	2030	6	**Fruit**	Outside and Cut	**yes**	**yes**

**Table 2:** Model Setup.

Model	Image Size	Weights Memory	Parameters	Depth	Datasets
	[px, px]	[MByte]			
EfficientNetB3	300, 300	47.6	12.3 M	210	2, 3
EfficientNetB4	380, 380	75	19.5 M	258	3
EfficientNetB5	456, 456	118	30.6 M	312	3
EfficientNetB3 Ensemble	300, 300	144	37.5 M	210	1

**Table 3:** Model Performance over 5 independent training runs.

Variant	Model	Dataset	Accuracy			Image Type
			Max	Average	Deviation	
v1	EfficientNetB3-Ensemble	ens	0.9424	0.9424		single
v2	EfficientNetB3	mixed	0.8333	0.8111	0.0223	single
v3	EfficientNetB3	3er	0.8849	0.8763	0.0079	combined
v4	EfficientNetB4	3er	0.9137	0.8791	0.0354	combined
v5	EfficientNetB5	3er	0.9065	0.8763	0.0372	combined
v6	EfficientNetB3	long	0.9137	0.8878	0.0231	single

## Data Availability

Data are contained within the article.
